# Thermal Degradation of Palm Fronds/Polypropylene Bio-Composites: Thermo-Kinetics and Convolutional-Deep Neural Networks Techniques

**DOI:** 10.3390/polym17091244

**Published:** 2025-05-02

**Authors:** Abdulrazak Jinadu Otaru, Zaid Abdulhamid Alhulaybi Albin Zaid

**Affiliations:** Department of Chemical Engineering, College of Engineering, King Faisal University, Al Ahsa 31982, Saudi Arabia

**Keywords:** palm fronds, polypropylene, thermal degradation, thermo-kinetic, machine learning

## Abstract

Identifying sustainable and efficient methods for the degradation of plastic waste in landfills is critical for the implementation of the Saudi Green Initiative, the European Union’s Strategic Plan, and the 2030 United Nations Action Plan, all of which are aimed at achieving a sustainable environment. This study assesses the influence of palm fronds (PFR) on the thermal degradation of polypropylene plastic (PP) using TGA/FTIR experimental measurements, thermo-kinetics, and machine learning convolutional deep learning neural networks (CDNN). Thermal degradation operations were conducted on pure materials (PFR and PP) as well as mixed (blended) materials containing 25% and 50% PFR, across degradation temperatures ranging from 25 to 600 °C and heating rates of 10, 20, and 40 °C·min^−1^. The TGA data indicated a synergistic interaction between the agricultural waste (PFR) and PP plastic, with decreased thermal stability at temperatures below 500 °C, attributed to the hemicellulose and cellulose present in the PFR biomass. In contrast, at temperatures exceeding 500 °C, the presence of lignin retards the degradation of the PFR biomass and blends. Activation energy values between 81.92 and 299.34 kJ·mol^−1^ were obtained through the application of the Flynn–Wall–Ozawa (FWO) and Kissinger–Akahira–Sunose (KAS) model-free methods. The application of CDNN facilitated the extraction of significant features and labels, which were crucial for enhancing modeling accuracy and convergence. This modeling and simulation approach reduced the overall cost function from 41.68 to 0.27, utilizing seven hidden neurons, and 673,910 epochs in 13.28 h. This method effectively bridged the gap between modeling and experimental data, achieving an R^2^ value of approximately 0.992, and identified sample composition as the most critical parameter influencing the thermolysis process. It is hoped that such findings may facilitate an energy-efficient pathway necessary for the thermal decomposition of plastic materials in landfills.

## 1. Introduction

The increasing necessity to produce bio-based, biodegradable, and compostable plastics utilizing sustainably sourced biomass, such as organic waste and residues (as opposed to primary biomass), is of significant interest to plastic manufacturers, environmental agencies, and policymakers worldwide. This initiative aims to reduce reliance on petroleum (fossil fuels) as the primary feedstock for plastic processing, within the framework of the circular economy as articulated in Saudi Vision 2030. This is particularly relevant to environmental protection initiatives, such as the Saudi Green Initiative, which is focused on the preservation of Saudi Arabia’s terrestrial and marine ecosystems. This approach emphasizes sustainable economic methods encapsulated in the principles of reduce, reuse, recycle, and remove (the 4Rs). Furthermore, this initiative aligns with the 2030 United Nations Sustainable Development Goals (Goal 12), which focus on waste reduction and reuse, as well as the European Union’s strategic plan on the efficient utilization of raw materials and energy conservation. The implementation of this initiative could facilitate the end-of-life conversion of bio-based plastics into smaller molecules (monomers) through enzymatic action secreted by microorganisms in landfill environments [1] or through energy conservation mechanisms such as thermal decomposition operations [2].

Analogous research studies focused on energy conservation and plastic degradation have incorporated agricultural waste as a component of the feedstocks, facilitating the thermal decomposition of non-biodegradable plastics within a thermogravimetric analyzer. The data derived from these thermogravimetric analyses (TGA) are subsequently utilized to quantify the kinetic and thermodynamic parameters vital for elucidating the reaction mechanism, energy consumption, and operational conditions of the process. For instance, Shagali et al. [3] investigated the effects of high heating rates (100, 500, and 1000 °C·min^−1^) on the photothermal thermogravimetric analysis of plastics (PET and PVC) and agricultural waste (corncob and peanut shell). The study revealed a synergistic interaction between the blended samples, particularly in the main decomposition region (250–500 °C), which led to a rapid release of volatiles. The researchers employed the Coats–Redfern model-fitting methods to identify the different reaction mechanisms for the various blends and utilized the FWO, KAS, and Starink (STK) model-free methods to estimate the activation energy and enthalpy for the blended samples, which ranged from 5 to 6 kJ·mol^−1^. Additionally, experimental TGA measurements in [4] demonstrated that the structural differences in biomass (cotton stalk, sunflower residue, hazelnut shell, and *Euphorbia rigida*) and plastics (PET and PVC) affect their thermal decomposition mechanisms and thermo-kinetic behaviors. Najafi et al. [5] examined the effect of the biomass (tea stem [TS])/PET ratio on the thermal decomposition of TS and PET using a thermogravimetric analyzer at various heating rates: 10, 15, 20, and 25 °C·min^−1^. The study concluded that a higher PET composition (75%) exhibited lower antagonistic effects compared to the biocomposite characterized by a higher TS composition (75%), which demonstrated strong synergistic positive and negative effects. A similar phenomenon was also reported in [6,7,8,9], highlighting the capacity of hemicellulose and cellulose in biomass to reduce the thermal stability of plastics, thus lowering the overall energy requirements for the process.

In contemporary research and industrial contexts, machine learning modeling and simulation tools have garnered substantial interest for their applications in predicting material compositions and optimizing thermal decomposition processes. For instance, Tian et al. [10] explored the applicability of one-dimensional convolutional neural networks paired with long short-term memory (1D-CNN-LSTM) algorithms within deep learning frameworks to predict thermogravimetric analysis (TGA) data derived from the combustion of fine screenings classified from municipal solid waste. The study [10] achieved a high coefficient of determination (R^2^) of 0.944 by employing a rigorous methodology that included 150 neurons, a dropout rate of 0.0003, 200 epochs, and a batch size of 25. Similarly, Alhulaybi and Otaru [11] applied machine learning techniques, specifically deep neural networks and regression modeling, to forecast the chemical stability and thermal properties of PLA/HKUST-1 fabricated porous membranes. The modeling approach in [11] was framed as a function of casting thickness, immersion time, and immersion temperature, with predictions aligning closely with experimental variations (i.e., 0.01–2.16% residual error). Furthermore, the research presented in [6] introduced a deep neural network (DNN) framework and learning algorithms that predicted the mass loss profile resulting from the thermal degradation of a bio-composite comprising *Phoenix dactylifera* L. and high-density polyethylene (HDPE) under heating rates of 10, 20, and 40 °C·min^−1^. This study reported significant enhancements in modeling responses, achieving a reduction in the overall cost function to 0.025 after just over a million epochs. Although numerous applications of machine learning tools have been employed to predict and optimize thermolysis operations [12,13,14,15], the use of convolutional neural networks (CNN) has predominantly been focused on imaging, with limited applications in the realm of thermal decomposition data. Consequently, this study provides a comprehensive assessment of the thermal degradation of palm fronds (biomass)/polypropylene 520L blends utilizing TGA/FTIR data, machine learning convolutional deep neural networks (CDNN), and their thermo-kinetic analysis.

While there is a substantial body of documented evidence regarding the thermal degradation of polypropylene (PP) and limited literature concerning the thermal degradation of palm fronds (PFR), it is essential to highlight that the utilization of this agro-waste material as a feedstock for the decomposition of polypropylene has not been reported to the best of the author’s knowledge. Additionally, Saudi Arabia hosts numerous petrochemical industries that are involved in the production of plastics for both domestic use and international exports, thereby contributing to the country’s gross domestic earnings. The increased production and utilization of these plastics have led to a rise in plastic waste within landfills. Furthermore, Saudi Arabia is rich in date palms, which are cultivated across various regions of the country, resulting in a significant availability of date waste and palm fronds. This study, for the first time, employs palm fronds as a feedstock for the thermal degradation of polypropylene, intending to understand the thermal decomposition of these materials and their composites under controlled conditions, as well as quantifying their kinetic and thermodynamic parameters. This research is anticipated to inform decisions regarding the potential use of palm fronds as a biocatalyst to accelerate the thermal degradation of end-of-life polypropylene (PP). Lastly, the application of machine learning, specifically convolutional deep neural networks (CDNN), for training and optimizing such data is intended to provide trained arbitrary constants capable of predicting the resulting thermograms measured during this process.

## 2. Experimental Procedure

Thermogravimetric analysis and Fourier transform infrared (FTIR) measurements were conducted to determine mass loss profiles and identify the molecular compounds present in the studied materials. The materials under investigation include powdered forms of palm fronds (also known as date leaf) and polypropylene. The palm fronds (PFR) were sourced from Al-Ahsa, located in the eastern region of Saudi Arabia, while the polypropylene 520L (PP) was obtained from Saudi Basic Industries Corporation (SABIC). Detailed information regarding the properties of the PP material can be found in [16].

Clean samples of the two materials, PFR and PP, were collected and oven-dried at 100 °C for 12 h. The weights of the samples prior to and following the drying operation were measured to determine the moisture content present within the samples. After drying, the samples were size-reduced into powders with particle sizes below 200 µm. A Cary 630 FTIR, equipped with Agilent MicroLab software (version 5.8), was used to measure the transmittance of infrared radiation for each sample across varying wavelengths. The thermal decomposition measurements followed a procedure like those described in [4,17,18,19]. Thermal decomposition of the samples was conducted using a Mettler Toledo TGA/SDTA 851e system, with temperatures ranging from 25 to 600 °C at three different heating rates: 10, 20, and 40 °C·min^−1^. Additionally, thermal degradation was performed on synthetic blends of the samples containing 25% and 50% PFR, under the same degradation temperatures and heating rates. To maintain an inert heating environment within the TGA system, nitrogen gas (N_2_) was introduced at a flow rate of 40 mL·min^−1^ before the thermal decomposition process commenced. This step was crucial to prevent oxidative mass loss from combustion during thermal decomposition operations. The sample was placed in the TGA heating chamber at 25 °C (room temperature), and the system was activated. Heating continued at a rate of 10 °C·min^−1^ until a maximum temperature of 600 °C was reached. At this point, complete degradation of the plastic material was expected, along with significant changes in mass. After reaching this temperature, the TGA system was turned off, and both the sample and the system were allowed to cool. The final mass of the sample was then measured. This procedure was repeated for each heating rate (10, 20, and 40 °C·min^−1^), various material compositions, and degradation temperatures, resulting in a total of twelve experimental measurements that were obtained and analyzed. Furthermore, the derivative thermogravimetric (DTG) data for this process were derived from the TGA data collected during the thermal decomposition process.

## 3. Analysis of TGA, DTG, and FTIR Data

**Figure 1:**
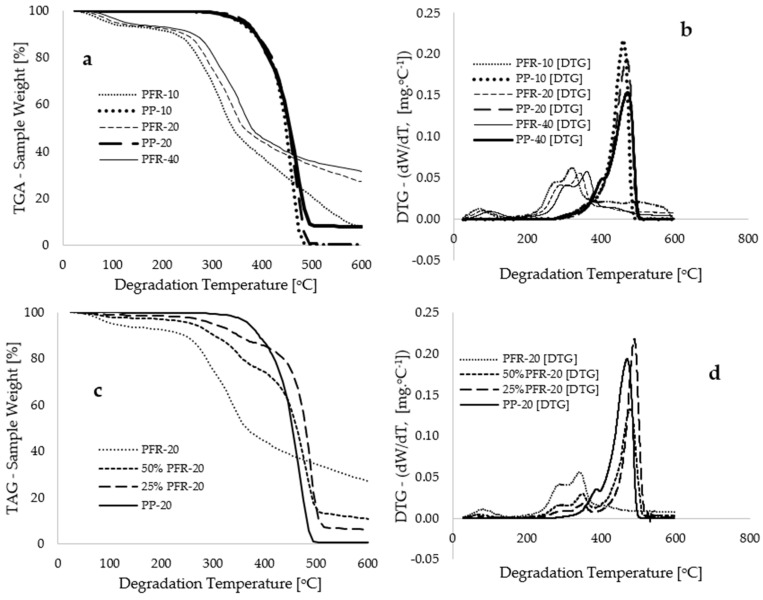
(**a**) Plots depicting the TGA weight percentage against degradation temperature (thermograms) for PFR and PP at varying heating rates of 10, 20, and 40 °C·min^−1^; (**b**) Derivative thermograms (DTG) for PFR and PP, at varying heating rates of 10, 20, and 40 °C·min^−1^; (**c**) Thermograms representing varying sample compositions at a constant heating rate of 20 °C·min^−1^; and (**d**) Derivative thermogravimetric representing varying sample compositions at a constant heating rate of 20 °C·min^−1^.

Figure 1 illustrates experimentally measured TGA and DTG data obtained for the selected samples. Figure 1a displays selected data points of percentage sample weight for the individual thermolysis of the PFR biomass and PP plastic at various heating rates (10, 20, and 40 °C·min^−1^) against degradation temperature (°C). A general observation regarding the thermal degradation of these materials is that increased weight loss corresponds with an elevation in degradation temperature, ranging from 25 °C to 600 °C. Additionally, a consistent shift in the thermograms toward higher temperature maxima was observed with increasing heating rates, a finding that aligns with previous studies [20,21,22,23]. Specifically, at a 50 wt% loss, degradation temperatures of 341.20, 364.27, and 379.19 °C were recorded for the PFR biomass at heating rates of 10, 20, and 40 °C·min^−1^, respectively. In the case of the PP plastic, degradation temperatures of 446.29, 454.51, and 463.44 °C were obtained at a 50 wt% loss for the corresponding heating rates. The raw experimental data indicated that the time required to reach the maximum degradation temperature of 600 °C varied according to the heating rate employed. Specifically, it took approximately 3450 s to conduct the TGA experiment at a heating rate of 10 °C·min^−1^, whereas approximately half and a quarter of this duration were necessary to attain the maximum degradation temperature of 600 °C for the samples subjected to heating rates of 20 and 40 °C·min^−1^, respectively.

Figure 1a illustrates the various thermal degradation stages for both materials: initiation, propagation, and termination stages. For polypropylene (PP) plastic, these stages occur within the temperature ranges of 25–305 °C, 305–485 °C, and 485–600 °C, respectively. The initiation stage is characterized by a temperature range in which thermal decomposition is primarily attributed to the loss of moisture content [24], accounting for less than 1.0% of the total moisture content [25]. The propagation stage signifies a temperature range where weight loss is reported [6] to be associated with the loss of material content, which constitutes nearly 90 wt% of the total weight loss. The derivative thermogravimetric (DTG) curves presented in Figure 1b demonstrate significant peaks at 461, 471, and 476 °C corresponding to heating rates of 10, 20, and 40 °C·min^−1^, respectively. These peaks offer essential insights into the maximum decomposition temperatures associated with the rapid degradation of the PP plastic material during the intermediate stage of the thermolysis process. Conversely, the termination stage of polypropylene degradation pertains to the residual ash content remaining after the thermal degradation of the sample. Huang et al. [26] identified a temperature range of 277–477 °C for the propagation (intermediate) stage during the thermal decomposition of polypropylene. In a separate experimental study, Esmizadeh et al. [27] recorded values of 325–495 °C for this intermediate stage of polypropylene thermal degradation, while Dobrzyńska-Mizera et al. [25] reported that polypropylene undergoes a single-step degradation, with the onset of the propagation stage beginning at 350 °C and concluding at 455 °C.

**Figure 2:**
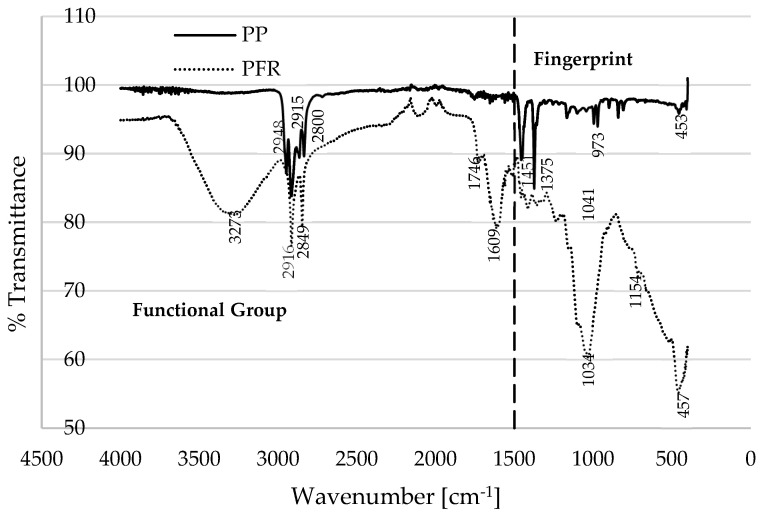
FTIR plots for PFR and PP, illustrating percentage transmittance as a function of wavenumber (cm^−1^).

The degradation of PFR biomass, as illustrated in Figure 1a, exhibits early degradation, a shift in the thermograms to lower temperatures during the intermediate stage, and a gradual degradation at elevated temperatures, in contrast to PP plastic. This behaviour may be attributed to the specific composition of the materials involved. Figure 2 presents Fourier transform infrared (FTIR) spectroscopy data for the two materials (PFR biomass and PP plastic), depicting typical plots of percentage transmittance against wavenumber (cm^−1^). The FTIR plots can be categorized into fingerprint (FP) and functional group (FG) or the diagnostic region [28]. According to the FTIR database in [29], the prominent peaks in the PP material, specifically, at 2800, 2916, and 2940 cm^−1^ are indicative of the O–H stretching of alcohol, the O–H stretching of carboxylic acid, and the C–H stretching of alkanes, respectively. Gao et al. [30] reported that the primary constituent of polypropylene is the alkane group (CH_2_–CHCH_3_), which tends to undergo thermal degradation like polyethylene (PE). Conversely, the characteristic peaks observed for PFR biomass can be assigned to the O–H stretching of alcohol (3273 cm^−1^), the O–H stretching of cellulose and hemicellulose (2916 cm^−1^), the C=O stretching of pectin and hemicellulose (1746 cm^−1^), and the C=C stretching of lignin (1609 cm^−1^). It is noteworthy that the wavenumbers below 1500 cm^−1^ in the FTIR plots are typically referred to as the fingerprint region [28], which is a region that is highly characteristic of the molecules as a whole; however, the spectrum within this region is generally considered less reliable for identification purposes [31].

Figure 2 illustrates that the transmittance of the PP plastic exceeds that of the PFR biomass, indicating a lower absorption of infrared radiation by the PP plastic material. Conversely, a higher proportion of this infrared radiation is absorbed by the PFR biomass materials [32]. Below 500 °C, Figure 1a demonstrates that the thermal stability of the PP plastic surpasses that of the PFR biomass. This distinction in thermogram characteristics among the samples may be attributed to the molecular compounds present in the materials, as well as their thermal conductivity. The O–H stretching associated with alcohol, hemicellulose, and cellulose in the PFR biomass indicates a high moisture content (6.7 wt% measured in this study), which could explain its earlier degradation when compared to the PP plastic material. The delayed degradation and higher thermal stability of the PP plastic, commencing at 360 °C, can be ascribed to its low moisture content (typically below 0.2 wt% as reported in [25]) and the significant proportion of alkanes (C–C bonds) contributing to the material’s elevated thermal conductivity [33]. Beyond 500 °C, as shown in Figure 1a, slow degradation is observed for the PFR biomass, while complete degradation to ash is evident for the PP plastic. For instance, at 555 °C, the mass compositions of PFR and PP recorded at a constant heating rate of 20 °C·min^−1^ are 30.13 wt% and 0.55 wt%, respectively. The slow degradation of PFR biomass at elevated temperatures may be attributed to the presence of C=C stretching associated with lignin in the materials [27]. Additionally, increasing the heating rate at these high temperatures further retards the decomposition of the lignin content in the biomass, as evidenced in Figure 1a. It is crucial to note that the DTG curves (Figure 1b) for the PFR biomass exhibit multiple peaks during the propagation stage, which can be attributed to the decomposition of hemicellulose, cellulose, and lignin at varying degradation temperatures. Experimental TGA measurements in [34,35,36] observed that the pyrolysis rate of biomass is directly proportional to its cellulose composition and inversely related to lignin content, suggesting that the decomposition of lignin represents a rate-limiting step in the process.

The differences in molecular composition and thermal stability exhibited by these two materials suggest that the formation of a biocomposite through the mechanical mixing of palm fronds (PFR) and polypropylene (PP) may impact the thermograms obtained for each individual material. Figure 1c,d presents the thermograms corresponding to the thermal decomposition of the blends at various material compositions and heating rates. These figures indicate that the consistent incorporation of PFR into the blend diminishes its thermal stability at lower temperatures, attributable to the increasing cellulose and hemicellulose contents within the biomass. The thermograms generated for the blends demonstrate a synergistic interaction between the biomass and plastic materials, leading to multi-step degradation that can be associated with the distinct degradation temperatures of hemicellulose and cellulose. El-Sayed et al. [37] reported that thermal degradation of hemicellulose and cellulose in palm fronds occurs within the temperature ranges of 220–300 °C and 300–340 °C, respectively. The current study, as illustrated in Figure 1a,b, reveals that at a heating rate of 20 °C·min^−1^, the degradation of hemicellulose and cellulose occurs at temperatures of 135–275 °C and 275–358 °C, respectively. Temperatures exceeding this range may be characterized as lignin degradation. Moreover, at degradation temperatures below 500 °C, the elevated amounts of hemicellulose and cellulose in the 50% PFR biomass sample contributed to its lower thermal stability when compared to the 25% PFR blend and pure PP plastic. Conversely, the 50% PFR sample is anticipated to possess a higher lignin content, which may account for its slower degradation at elevated temperatures, typically above 500 °C. For example, at 505 °C and 600 °C, the compositions for the 25% PFR materials were 10.36 and 6.14 wt%, respectively, while higher values of 13.85 and 10.80 wt% were observed for the 50% PFR material at the corresponding degradation temperatures. Consequently, the alterations in the thermograms produced by the mechanical mixtures are expected to also influence their resulting thermo-kinetics under varying heating rates and degradation temperatures.

## 4. Kinetics and Thermodynamics

The kinetics and thermodynamics of thermal decomposition processes were investigated to elucidate potential reaction mechanisms and to ascertain the kinetic and thermodynamic parameters involved. The kinetic parameters under consideration include activation energy ($E_{A}$) and the pre-exponential factor (*A*), while the thermodynamic parameters encompass changes in enthalpy (∆H), entropy (∆S), and Gibbs free energy (∆G). The reduced theoretical (Equation (1)) and experimental (Equation (2)) Criado master plots [36,38] in conjunction with selected solid-state reaction mechanisms (Table 1) were initially utilized to identify the reaction mechanism(s) that most accurately characterize the thermal degradation process. This analysis preceded the selection of model-fitting approaches, model-free kinetic models, or the two models used for the estimation of the thermo-kinetic parameters associated with the process.

**Criado Master Plots**

(1) $$\mathrm{Reduced}\;\mathrm{Theoretical}\;\mathrm{Equation}:\;\frac{Z(x)}{Z(0.5)}=\frac{f\left(x\right)\cdot g(x)}{f\left(0.5\right)\cdot g(0.5)}$$

(2) $$\mathrm{Reduced}\;\mathrm{Experimental}\;\mathrm{model}:\;\;\frac{Z(x_{i})}{Z(0.5)}={\left(\frac{T_{x}}{T_{0.5}}\right)}^{2}\frac{{\left(dx/dt\right)}_{x}}{{\left(dx/dt\right)}_{0.5}}$$

where 0.5 refers to the conversion at $x_{i}$ = 0.5 and $x_{i}=\frac{w_{o}-w_{t}}{w_{o}-w_{f}}$.

**Coats–Redfern (CR) Model-Fitting Method [38,39,40]:**

(3) $$\ln\left[\frac{g(x_{i})}{T^{2}}\right]=\ln\left(\frac{A\cdot R}{Q_{R}\cdot E_{A}}\right)-\left(\frac{E_{A}}{R}\right)\cdot \frac{1}{T}$$

**Figure 3:**
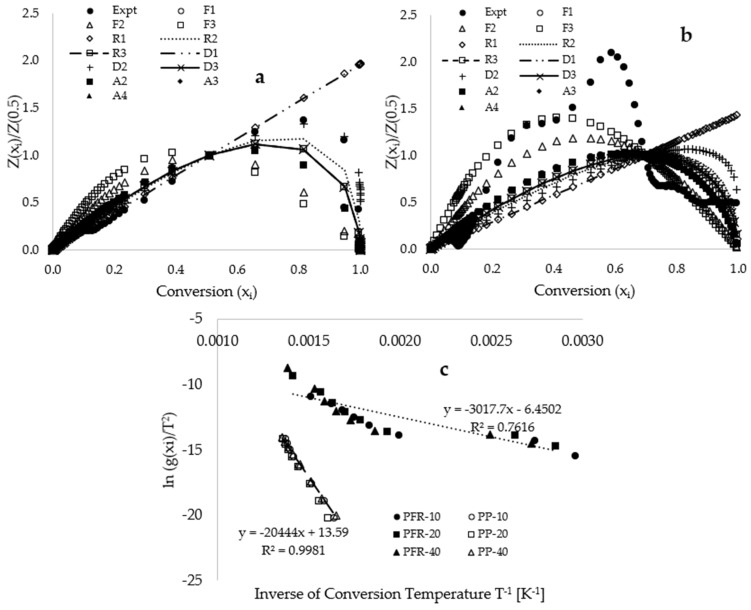
Criado master plots displaying the relationships between reduced theoretical and measured $\left[Z\left(x_{i}\right)/Z\left(0.5\right)\right]$ data against conversion ($x_{i}$) for (**a**) PP plastic, and (**b**) PFR biomass samples, and (**c**) plots of Coats–Redfern’s ln g(xi)/T^2^ against the inverse of conversion temperature for both the PP and PFR samples.

Figure 3 presents the Criado master plots of both the reduced experimentally measured and theoretical data against conversion for the selected well-known solid-state reaction mechanisms utilized in kinetic studies of the thermal degradation of materials. Figure 3a indicates that the application of these master plots suggests that the degradation of polypropylene (PP) may follow either the geometrical contraction model (contracting cylinder [R3]) or the three-dimensional diffusion model (Jander [D3]). This observation implies that the implementation of these reaction models in model-fitting methodologies for estimating kinetic and thermodynamic parameters for PP plastic could yield higher coefficients of determination. Research documented in [41] identified a similar contracting cylinder model as the estimated solid-state reaction mechanism for polypropylene plastic. In contrast, Figure 3b reveals a poor fit between the reduced experimentally measured plot and the corresponding reduced theoretical curves, particularly at both higher and lower conversions, for the PFR biomass sample. This finding suggests that the potential applicability of model-fitting methods may lead to low coefficients of determination and could consequently result in significant variations in activation energy across the range of conversions. The recommendation by Vyazovkin et al. [42] at the International Conference on Thermal Analysis and Calorimetry suggests that substantial variation in activation energy along the conversion line necessitates the selection of a model-free method (to be discussed) as the appropriate kinetic model. However, the application of the model-fitting kinetic method may potentially result in significant errors in the estimates of activation energy [42]. Figure 3c presents plots of Coats–Redfern’s (Equation (3)) ln g(x_i_)/T^2^ against the inverse of conversion temperature for both the PP and PFR samples, across varying heating rates of 10, 20, and 40 °C·min^−1^. This figure indicates a poor fit of linear inverse trends when the third-order reaction model [F3] is employed as the selected solid-state reaction mechanism, as it was the closest to the reduced experimental curve observed in the Criado master plots in Figure 3b. Consequently, the selection of a model-free kinetic method for variable heating rates is warranted for the estimation of kinetic and thermodynamic parameters associated with this process.

The non-isothermal Flynn–Wall–Ozawa (Equation (4)) and Kissinger–Akahira–Sunose (Equation (5)) model-free methods, which are applicable to variable heating rates, were selected for the estimation of the kinetic and thermodynamic parameters associated with this process. Furthermore, the mathematical expressions employed for the estimation of the thermodynamic parameters [40,43] are presented by Equations (6)–(8).

**Model-Free Iso-Conversional Methods for Multiple Heating Rates**

Flynn–Wall–Ozawa (FWO) Model [40,43]

(4) $$ln[Q_{R}]=ln\left(\frac{A\cdot E_{A}}{R\cdot \left(g\left(x_{i}\right)\right)}\right)-5.331-1.052\left(\frac{E_{A}}{R}\right)\cdot \frac{1}{T}$$

Kissinger–Akahira–Sunose (KAS) Model [40,43]

(5) $$ln[Q_{R}/T^{2}]=\ln\left(\frac{A\cdot R}{E_{A}\cdot \left(g\left(x_{i}\right)\right)}\right)-\left(\frac{E_{A}}{R}\right)\cdot \frac{1}{T}$$

**Thermodynamics Properties**

(6) $$∆H=E_{A}-R\cdot T_{M}\;(\mathrm{change}\;\mathrm{in}\;\mathrm{Enthalpy})$$

(7) $$∆S=R\cdot \ln\left(\frac{A\cdot h}{K\cdot T_{M}}\right)\;(\mathrm{change}\;\mathrm{in}\;\mathrm{Entropy})$$

(8) $$∆G=∆H-T_{M}\cdot ∆S\;(\mathrm{change}\;\mathrm{in}\;\mathrm{Gibbs}\;\mathrm{Free}\;\mathrm{Energy})$$

where $E_{A}$ is the activation energy (kJ·mol^−1^), R is the gas constant in J·mol^−1^·K^−1^, $Q_{R}$ denotes the heating rate (°C·min^−1^), $h=6.626\times 10^{-34}\;J\cdot s^{-1}$ represents Planck’s constant, K = 1.3806 × 10^−23^ J·K^−1^ is the Boltzmann constant, and $T_{M}$ signifies the final or maximum temperature of constant conversion. It is important to recognize that both model-fitting and model-free methods have their own merits and demerits. While model-free methods are often regarded as providing a superior approximation of kinetic parameters [38,44], their application typically necessitates the use of TGA measurements at a minimum of three different heating rates [42]. In contrast, the model-fitting method requires only a single heating rate, which can also facilitate the estimation of potential reaction mechanisms for the process [40]. However, Figure 3c revealed that the application of the Coats–Redfern model-fitting kinetic methods resulted in a decreased coefficient of determination due to non-linear deviations in the data points at lower temperatures [38], complicating the estimation of kinetic parameters. A careful examination of Figure 1a reveals that the PP plastic material completely decomposes to ash just before reaching a degradation temperature of 500 °C. Beyond this temperature, the lignin content in the PFR biomass inhibits further degradation. Consequently, the Criado master plots presented in Figure 3a demonstrate that both model-fitting and model-free methodologies can be utilized to extract kinetic parameters for PP plastics up to 90 wt% conversion. However, Figure 1a illustrates that employing model-free methods on thermograms obtained from the PFR and its blends for conversions exceeding 60 wt% loss may yield data only for thermograms conducted at a heating rate of 10 °C·min^−1^, which is insufficient for the application of multiple heating rate kinetic models [44]. Therefore, the kinetic and thermodynamic data for thermal decomposition operations were obtained for conversions ranging from 2.5 to 60.0 wt% loss.

**Figure 4:**
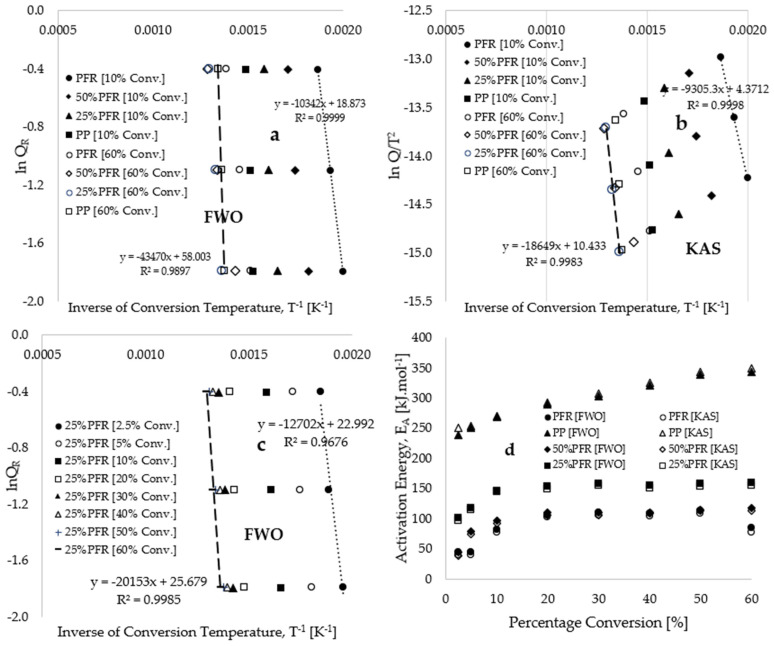
Plots of (**a**) estimated lnQ_R_ using the FWO method against the inverse of conversion temperature, T^−1^ [K^−1^], (**b**) estimated ln[Q_R_/T^2^] using the KAS method against the inverse of conversion temperature, T^−1^ [K^−1^], (**c**) estimated lnQ_R_ using the FWO method against the inverse of conversion temperature, T^−1^ [K^−1^] for the 25% PFR sample at conversions ranging between 2.5 and 60 wt%, and (**d**) estimated activation energy using the FWO and KAS methods against percentage conversion [%].

Figure 4 presents kinetic trend plots estimated using the model-free methods of Flynn–Wall–Ozawa (FWO) (Equation (4)) and Kissinger–Akahira–Sunose (KAS) (Equation (5)). Table 2 provides calculated values of kinetic and thermodynamic data derived from both the application of FWO and KAS model-free methods. Figure 4a displays plots of FWO’s lnQ_R_ against the inverse of the conversion temperature for various sample compositions at 10 wt% and 60 wt% conversions. Corresponding plots utilizing the KAS model-free method are depicted in Figure 4b. Both figures illustrate linear inverse trends with an increase in the inverse conversion temperature, which shifts to temperature maxima with increasing plastic composition in the blends and with rising conversion (i.e., mass loss due to material decomposition). The high values of the estimated coefficient of determination (R^2^~1.0) for the plots in Figure 4 indicate the robust applicability of the multiple heating rates model-free method in estimating the kinetic and thermodynamic parameters of this process, particularly when compared to single heating rate model-fitting kinetic method. Figure 4c presents FWO’s lnQ_R_ plotted against the inverse of conversion temperature for a constant sample composition and heating rate (25% PFR-20) across varied conversions ranging from 2.5 to 60 wt%. This indicates a consistent shift of the trends towards temperature maxima at higher conversions and towards temperature minima at lower conversions, findings that are consistent with those reported in [45,46]. Notably, Figure 4c indicates that the linear inverse trends at 2.5 to 20 wt% conversions are significantly dispersed in the low-temperature region, suggesting a substantial change in activation energy (see Figure 4d) resulting from the dehydration and decomposition of the cellulose and hemicellulose content present in the PFR biomass blends.

It is crucial to clarify that a notable observation in any of the linear inverse trends depicted in Figure 4 is the consistent shift of data points towards temperature maxima, which occurs with increasing heating rates and increasing plastic composition in the composite samples. The increasing plastic composition in the biocomposites led to a rise in petrochemical content as well as an enhancement in the thermal stability of the sample [47]. Jin et al. [48] observed the synergistic interaction in the thermolysis of biomass and plastic and concluded that the increased wax formation at elevated temperatures can be attributed to the alkenes and alkanes present in PP plastic. Moreover, the elevated oxygen and hydrogen content in the biomass has reportedly resulted in the early formation of gas at lower temperatures [48,49] and slow degradation to ash at higher temperatures [25]. Figure 4d and Table 1 demonstrate that the estimated values of activation energy ($E_{A}$) obtained using the two model-free kinetic methods exhibit close agreement, characterized by high coefficients of determination. Specifically, Figure 4d illustrates that an increase in conversion rates and plastic composition within the blends results in an elevation of activation energy ($E_{A}$), particularly at lower temperatures (below 500 °C). For example, at conversion rates between 2.5% and 60% by weight, the estimated $E_{A}$ values for the PFR biomass and PP plastics, as determined by the FWO model-free method, ranged from 44.82 to 113.35 kJ·mol^−1^ and 238.72 to 343.55 kJ·mol^−1^, respectively. Similarly, estimated $E_{A}$ values for comparable conversions and materials using the KAS model-free method ranged from 40.46 to 108.73 kJ·mol^−1^ and 251.13 to 349.17 kJ·mol^−1^, respectively. These significant variations in activation energy, exceeding 20%, indicate that the degradation processes of both the PFR biomass and PP plastic are multi-step in nature, particularly when compared to the ICTAC recommendations reported by Vyazovkin et al. [42]. The DTG data presented in Figure 1 illustrate the multiple degradation steps observed during the propagation stages of the thermal degradation of these materials. Notably, the degradation of PFR biomass exhibited more pronounced multi-step behaviour, while such behaviour for the PP plastic was only detectable at a high heating rate of 40 °C·min^−1^. In terms of overall averages, Table 2 indicates that the estimated $E_{A}$ values for PFR biomass and PP plastics obtained via the FWO model-free methods are 86.54 and 294.14 kJ·mol^−1^, respectively. Moreover, for the 25% PFR sample, the estimated $E_{A}$ value was notably higher (143.41 kJ·mol^−1^) compared to the value obtained for the 50% PFR sample (97.84 kJ·mol^−1^), suggesting a greater energy requirement for the decomposition of increased plastic content in the biocomposites.

In comparison with the existing literature, El-Sayed et al. [37] reported activation energy values of 91.9 and 87.5 kJ·mol^−1^ for palm fronds, utilizing the FWO and KAS methods, respectively. Raza et al. [50] determined activation energy values ranging from 96 to 98 kJ·mol^−1^ for the thermal decomposition of palm surface fiber, identifying two distinct mass loss regions at temperatures of 270–390 °C and 390–600 °C. Aboulkas et al. [36] reported activation energy values between 179 and 188 kJ·mol^−1^ for the thermal decomposition of polypropylene plastic, derived from the FWO, KAS, and Starink (STK) methods, while Kartik et al. [51] obtained an average activation energy value of 224 kJ·mol^−1^ in separate experimental measurements. Additional estimated values for polypropylene in the literature [40,52,53,54] range from 102 to 327 kJ·mol^−1^, with the variability largely attributed to differences in polypropylene grade (notably changes in molecular weight), as well as the selection of heating rates and kinetic models. Furthermore, Table 2 indicates that the pre-exponential factor increases with an increasing plastic composition, suggesting that the degradation of hydrocarbon components in the plastic into monomers facilitates rapid chemical reactions, which result from high molecular collisions, disintegration, cleavage, and structural rearrangement at elevated degradation temperatures [47,55]. Notably, both the changes in enthalpy and Gibbs energy remain positive, indicating that the process is endothermic and non-spontaneous. Additionally, the change in entropy data consistently approaches zero across all selected kinetic models and chosen operating conditions for thermal decomposition operations, suggesting that the process is characterized by a lower degree of disorder [40,41].

## 5. Machine Learning Convolutional Deep Neural Networks (CDNN)

The modeling and simulation of the thermal degradation operations were accomplished using two advanced deep learning techniques within the realm of machine learning. This approach integrates convolutional neural networks (CNN) and deep neural networks (DNN) for data mining and predictions [56]. The methodology involves training and predicting the fraction of sample weight loss (output) as a function of input parameters, specifically heating rate (Q), degradation temperature (T), time (t), and sample composition (C). The rationale for employing the CNN modeling technique in this study is to enable the selected experimental data points to adaptively learn complex power structures through the processing of both input (feature) and output (label) data across a multilayer perceptron (MLP) comprising multiple filters or kernels [57,58]. This approach may enhance the selection of critical experimental data points that can function as features for the DNN—hence, the designation convolutional deep neural networks (CDNN).

Before the machine learning modeling and simulation, data preprocessing was performed on the raw experimental data. Initially, twelve experimental datasets containing 936 key data points were selected from the 24,152 raw TGA experimental data points. Fraction of mass loss, degradation temperature, and time were selected using an interval of 10 °C at both the initiation and termination stages of each thermogram, while a 5 °C interval was used at the intermediate (propagation) stage, which was considered important due to the significant weight loss occurring in a short time frame at this stage of degradation. Log normalization was applied to both the input (features) and output (labels) data, converting them into values between 0 and 1. This step is essential in machine learning because of the choice of the activation function (to be discussed later), which is critical for formulating learning algorithms and training the experimental data to identify more generalized patterns. This normalization was accomplished by dividing both features and labels by their respective maximum possible values: 100 °C·min^−1^ for heating rate, 1000 °C for degradation temperature, 7200 s for time, and 1.00 for the fractional composition of PFR in the blends.

**Figure 5:**
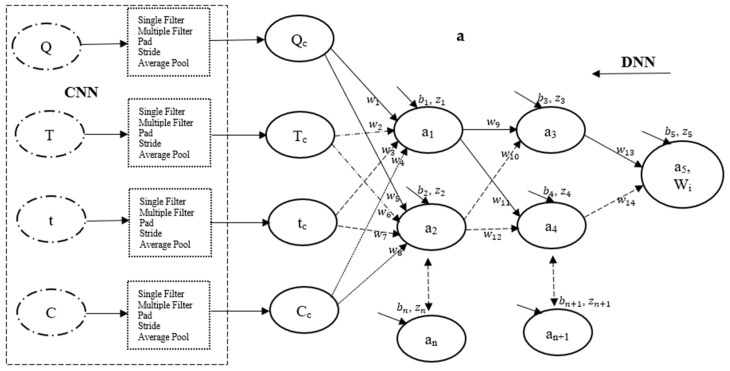

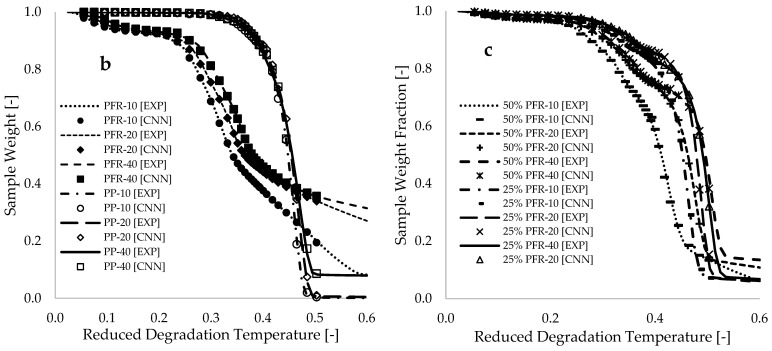
(**a**) A CDNN framework illustrating typical inputs, filters/kernels, hidden neurons, and an output neuron, and plots depicting the experimental and CNN-filtered sample weight fraction against reduced degradation temperature [-] provided for the (**b**) pure PFR and PP samples, as well as (**c**) 25% and 50% PFR/PLA blends.

To the left of Figure 5a, a convolutional neural network (CNN) framework is depicted, illustrating typical features, multilayer perceptron (filters), and labels. The output generated by the CNN framework is subsequently input into a structured deep neural network (DNN) framework (Figure 5a) for model training. This segment of the CNN framework demonstrates the progression of the input vector through a 3 × 3 arrangement of multiple filters comprising values of −1 and +1, resulting in three distinct vectors with values ranging between −1 and +1. It is crucial to recognize that these filters function as feature extractors, identifying silence in potential locations; this operation, executed overall positions, is termed convolution [58,59]. The three vectors produced by the initial kernels are then processed through another set of 3 × 3 filters in a single pass, yielding an output vector with values ranging from −1.00 to −0.07. A one-dimensional (1D) padding technique is applied to the output by introducing zeros at both boundaries, followed by the implementation of a 1D filter to ensure the preservation of classification dimensionality. Additionally, a two-step stride and average pooling operation are subsequently employed to further diminish the dimensionality of the final classification, resulting in a vector with values between 0 and 1. For a single entry of the dataset into the CNN framework, a total of 33 data points were extracted from 78 data points, culminating in approximately 396 data points extracted for each of the input and output parameters. Figure 5b,c present plots of sample fraction versus reduced degradation temperature for pure materials and blends, respectively. These plots demonstrate a complete overlap between the CNN-modeled data and the selected experimental data, with coefficients of determination exceeding 0.998 for all CNN modeling and simulation operations.

The data obtained using CNN techniques were integrated into a deep neural network (DNN) framework consisting of four input neurons, one output signal, and two hidden layers, each initially containing five neurons (see Figure 5a). The learning algorithms for this framework were developed based on general artificial neural network (ANN) models, as outlined in Equations (9)–(11). The learning algorithms employed in this study were developed following analogous to that reported by this group in [11,36,60]. Equation (9) represents the Sigmoid activation function (a), defined as the logistic function ($\sigma^{\prime }[z]$) of the sum of weight (Z) [61,62]. This sum weight is mathematically expressed as a function of synaptic weight ($w_{i}$), input parameters ($x_{i}$), and bias ($b_{i}$), as shown by Equation (10). Equation (11) details the cost function, represented as the residual error between the measured output and the output activation function, or predicted data. Notably, the choice of the Sigmoid activation function (a) in the CDNN framework illustrated in Figure 5a is due to its effectiveness in accurately capturing behaviours characterized by predicted responses ranging from 0 to 1 [61].

(9) $$a=\sigma^{\prime }[z]=\frac{1}{1+e^{-z}}$$

(10) $$Z=\sum_{i}w_{i}\cdot x_{i}+b_{i}$$

(11) $$C={\left(y-a\right)}^{2}$$

**Figure 6:**
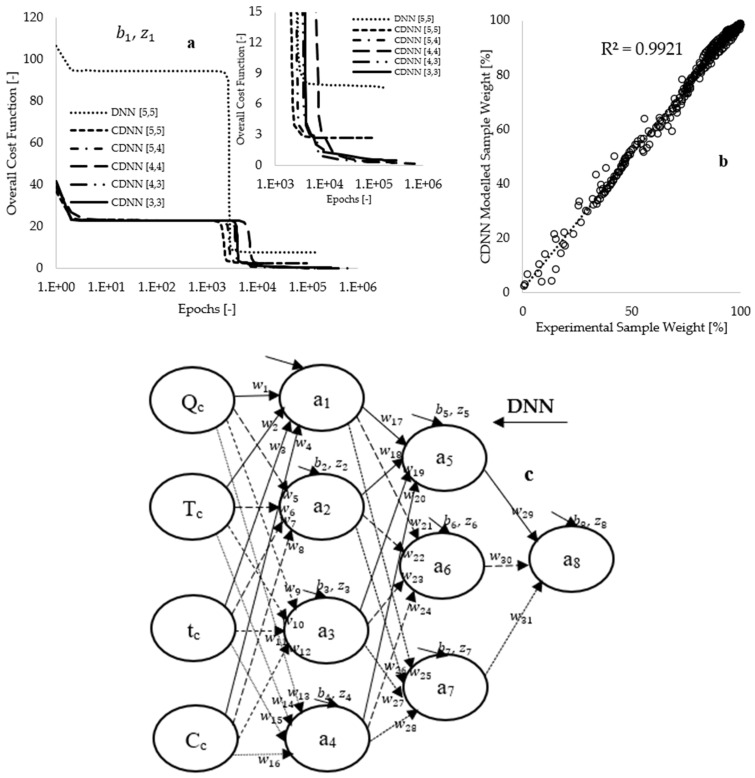
(**a**) Plots of the overall cost function against number of iterations (epochs), (**b**) the CDNN modeled sample weight [%] against experimental sample weight [%], and (**c**) the optimized DNN framework.

The formulated learning algorithms that incorporate cost optimization models were designed to identify patterns within the experimental measurements. This training was executed in Microsoft Excel (version 2016), supplemented by custom code written in Visual Basic for Applications (VBA). An initial computational time of one second was selected for the looping interval at the onset of the training, which was subsequently reduced to zero to expedite the training process. The objective of the training was to identify optimal arbitrary constants (i.e., synaptic weights and biases) that minimize the discrepancy between the model and experimental data, thereby reducing the overall cost function to nearly zero [60,62]. As illustrated in Figure 6a, the preliminary training conducted on the DNN[5,5] framework, utilizing 936 selected data points for each input parameter, resulted in an overall cost function of 106.32, which decreased to 7.59 upon completion of the training process. This approach reflects a reduction in true error to 7.14%, achieved over 161,597 epochs (loops) within a duration of 5.78 h. Subsequent training utilized 396 CNN-mined data points for each input fed into the CDNN[5,5] framework, which yielded an initial cost function of 36.94, declining to 2.70 after the training process, accomplished through 94,549 epochs in 2.77 h.

The approach of utilizing CDNN-mined data as input to the neural network may have resulted in a reduction in training time, effectively making the model smaller and faster through pruning. However, the true error value obtained with the CDNN[5,5] framework (7.30%) is nearly equivalent to the 7.14% achieved with the DNN[5,5] framework. Consequently, hyperparameter modeling was performed by systematically adjusting the learning rate from 5 to 2 during the training period, followed by a decrease in the number of hidden neurons within the architecture. Figure 6a illustrates that this approach led to the development of several CDNN frameworks, demonstrating a gradual reduction in the number of hidden neurons from 10 to 6. Optimal values for synaptic weights and biases were attained when the CDNN-modeled data completely overlapped with experimental measurements, as depicted in Figure 6b. Additionally, Figure 6a indicates that the CDNN[4,3] framework, which comprises seven hidden neurons, reduces the overall cost function from 41.68 to 0.27 (corresponding to a 0.64% true error), achieved after 94,549 epochs over a duration of 2.73 h.

Model regularization was implemented to mitigate modeling complexity, thereby achieving a balance between underfitting and overfitting while promoting the model’s ability to learn a more generalized pattern [6,57]. This was accomplished by extending the modeling duration by an additional 10.55 h, which further decreased the overall cost function to 0.19 (equating to 0.45% in true error), utilizing approximately 673,910 epochs in total, as illustrated in Figure 6a. Figure 6b demonstrates a complete overlap between modeling and experimental measurements, with an enhanced coefficient of determination of 0.992, suggesting that the model accurately learns the patterns associated with the experimental data. Figure 6c presents the newly developed CDNN[4,3] framework, while Table 3 displays the CDNN-computed data corresponding to this framework. A sensitivity analysis conducted using the data available in Table 3 indicates that higher synaptic weights are associated with the connections between the sample composition (Cc) and the first hidden layer. This finding suggests that sample composition is the most significant parameter influencing the thermal decomposition process, followed by the heating rates (Q) employed during the thermal degradation processes.

**Figure 7:**
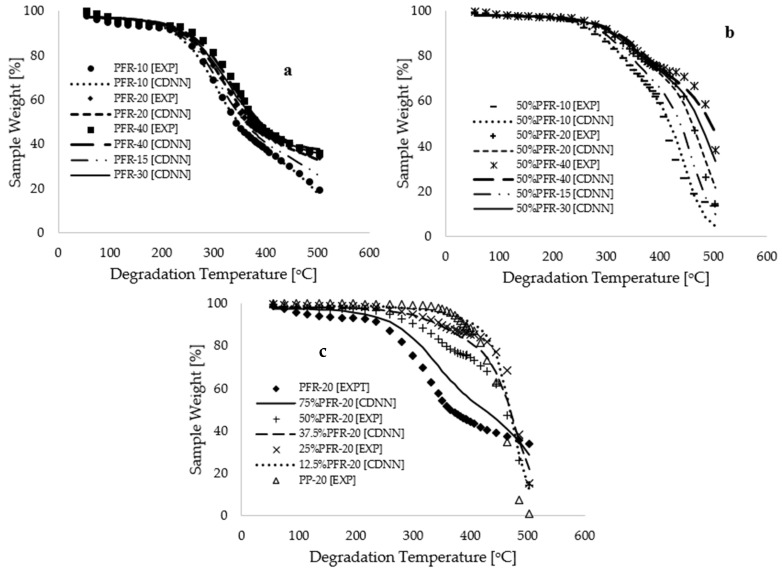
Plots of experimental and CDNN modeled sample weight [%] against degradation temperature [°C] for (**a**) pure PFR samples subjected to varied heating rates [°C·min^−1^], (**b**) 50% PFR samples examined at varied heating rates [°C·min^−1^], and (**c**) a constant heating rate of 20 °C·min^−1^ with varied sample compositions.

Validation and cross-validation of the model were conducted to evaluate its reliability by assessing its performance on the measured thermograms. Figure 7a,b illustrate that both the experimental and CDNN-modeled thermograms completely overlap for various operational conditions, including degradation temperatures, heating rates, and sample compositions. Although Figure 6b demonstrates a higher coefficient of determination (R^2^~0.992) between the measured and predicted data, the average values of the mass loss, along with their estimated uncertainties, for both experimentally measured and CDNN-predicted values are 78.44 ± 1.22% and 78.65 ± 1.20%, respectively. Furthermore, the estimated values of the mean bias errors (MBE) for both CDNN model predictions and experimental measurements are −5.55 × 10^−14^ and −1.49 × 10^−14^, respectively. Figure 7c shows that interpolated thermograms at different 12.5%, 37.5%, and 75% PFR biomass compositions fall within the expected limits of experimental measurements, thereby justifying a high level of modeling confidence. It is important to note that the kinetic analysis conducted in this study utilized data for conversion between 2.5% and 60% weight, considering degradation temperatures ranging from 64.12 to 498.99 °C. The application of a CNN deep learning approach in machine learning facilitated the extraction of features and labels corresponding to degradation temperatures ranging from 55.00 to 503.33 °C. This indicates that the kinetic and thermodynamic analyses for this process, utilizing the CDNN output data, remain consistent with the experimental data points initially collected for degradation temperatures ranging from 25 to 600 °C.

It is important to emphasize that the proposed convolutional deep neural network (CDNN) model accurately captured the behaviour characterized by the experimentally measured thermograms under various operating conditions selected for the process. Both the experimental measurements and CDNN predictions revealed the dependence of thermogravimetric analysis (TGA) curves on heating rates, degradation temperature, time, and sample compositions. Thermo-kinetic analysis of the experimental measurements indicates that the continued addition of PFR biomass feedstock into the blend consistently reduces the activation energy, pre-exponential factor, activation enthalpy, and Gibbs free energy. This approach created a more complex multi-step degradation at the propagation stage of the thermal degradation process, suggesting synergistic interactions between the selected materials. Moreover, the trained CDNN model and thermo-kinetic analysis presented in this study could provide valuable insights for environmental engineers and energy analysts regarding energy utilization and conservation during the end-of-life thermal degradation of polypropylene (PP) plastics in a landfill. Additionally, this approach could facilitate the potential utilization of PFR biomass as a bio-catalyst for the design of bio-based polypropylene plastics with enhanced thermal properties. However, such a study would require the formulation of PFR-PP composites, followed by analyses involving chemical stability, tensile and compressive strength measurements, density, melting point, durability, thermal and electrical conductivity, and reactivity tests. Furthermore, the role of thermal contact surfaces, specifically varying particle sizes, and commercial zeolite catalysts in the thermal degradation of polypropylene (PP) plastics warrants further investigation by this research group. This exploration could pave the way for subsequent studies involving the application of machine learning tools to predict the kinetics and thermodynamics associated with the thermal degradation of biomass–plastic composites.

## 6. Conclusions

The study investigates the thermal degradation of palm fronds (PFR) biomass and polypropylene (PP) plastic through the application of TGA/FTIR measurements, thermo-kinetic analyses, and convolutional deep neural networks (CDNN). The FTIR data obtained from these materials indicated the presence of hemicellulose, cellulose, pectin, and lignin constituents within the PFR biomass. In contrast, PP plastic is characterized by the presence of petrochemical compounds, primarily alkanes, with minor quantities of alcohol and carboxylic acids. The thermal decomposition process demonstrated that the presence of hemicellulose and cellulose in the PFR biomass contributed to a reduction in the thermal stability of the plastic at temperatures below 500 °C, while the lignin content in the PFR biomass inhibited degradation at elevated temperatures, typically exceeding 500 °C. Overall, the thermal degradation process revealed a synergistic interaction between these two materials, forming biocomposites that facilitated additional degradation steps during the propagation stage in the resulting thermograms.

The application of Criado master plots, in conjunction with twelve selected solid-state reaction mechanisms, indicated that the thermolysis of polypropylene (PP) plastic materials could adhere to either the geometrical contraction model (contracting cylinder [R3]) or the three-dimensional diffusion model (Jander [D3]). However, the Criado master plot demonstrated a poor fit between the reduced theoretical curves and the experimental curve for the PFR biomass at both low and high conversions. This finding suggests the inadequacy of model-fitting kinetic methods for estimating thermo-kinetic data in this process. To address this issue, the Flynn–Wall–Ozawa (FWO) and Kissinger–Akahira–Sunose (KAS) model-free methods were employed to estimate the thermo-kinetic parameters. These methods yielded a higher coefficient of determination for all sample compositions (that is, $R^{2}$ values ranging from 0.969 to 0.995). The application of these methods yielded activation energy ($E_{A}$) values ranging from 86.54 to 294.14 kJ·mol^−1^ and 81.92 to 299.34 kJ·mol^−1^ using the FWO and KAS methods, respectively, both consistently increasing with the rise in conversion and the PP plastic composition in the biocomposites. The overall change in enthalpy was found to range from 77.25 to 293.55 kJ·mol^−1^, while the change in Gibbs free energy varied from 163.67 to 207.04 kJ·mol^−1^, remaining positive throughout, which suggests that the thermolysis operations are endothermic and non-spontaneous, respectively. Additionally, the estimated values for the overall change in entropy, which ranged from −0.16 to −0.07 kJ·mol^−1^·K^−1^, were close to zero, indicating that the thermal decomposition process is characterized by low disorder.

The application of convolutional deep neural networks (CDNN) in this study facilitated the extraction of key features and labels essential for training the models. This methodology contributed to minimizing the model’s true error to 0.45%, achieved through 673,910 epochs and seven hidden neurons over a duration of 13.277 h. The predicted data obtained after modifications to the modeling process, which included hyperparameter tuning and regularization, exhibited a complete overlap with experimental results, yielding an overall coefficient of determination ($R^{2}$) of 0.992. Cross-validation was performed, leading to the determination of interpolated thermograms and activation energies that fell within the expected limits of experimental scatter, thereby indicating that the formulated learning algorithms have captured a more generalized pattern. Furthermore, the optimized synaptic weights and biases derived from the CDNN modeling and simulation identified the sample composition as the most significant parameter influencing the co-thermolysis of palm fronds and polypropylene, followed by variations in the heating rate employed.

## Figures and Tables

**Table 1:** Selected solid-state reaction mechanisms [37].

Reaction Mechanisms	$g\left(x_{i}\right)$	$f\left(x_{i}\right)$
First order reaction model [F1]	$-ln\left(1-x_{i}\right)$	$\left(1-x_{i}\right)$
Second order reaction model [F2]	${{(1-x}_{i})}^{-1}-1$	${{(1-x}_{i})}^{2}$
Third order reaction model [F3]	$\left[{{(1-x}_{i})}^{-2}-1\right]/2$	${{(1-x}_{i})}^{3}$
Geometrical contraction model (one-dimension [R1])	$x_{i}$	1
Geometrical contraction model (contracting sphere [R2])	${{1-(1-x}_{i})}^{1/2}$	${{2(1-x}_{i})}^{1/2}$
Geometrical contraction model (contracting cylinder [R3])	${{1-(1-x}_{i})}^{1/3}$	${{3(1-x}_{i})}^{2/3}$
One-dimensional diffusion model (D1)	${x_{i}}^{2}$	$1/2x_{i}$
Two-dimensional diffusion model (D2)	[$\left(1-x_{i}\right)ln\left(1-x_{i}\right)]+x_{i}$	${{[-ln(1-x}_{i})]}^{-1}$
Three-dimensional diffusion model (Jander [D3])	${\left[{{1-(1-x}_{i})}^{1/3}\right]}^{2}$	$3{{(1-x}_{i})}^{2/3}/[2\left({{1-(1-x}_{i})}^{1/3}\right)]$
Avarami-Erofe’ve (A2)	${{[-ln(1-x}_{i})]}^{1/2}$	${{2\left(1-x_{i}\right)[-ln(1-x}_{i})]}^{1/2}$
Avarami-Erofe’ve (A3)	$[{{-ln(1-x}_{i})]}^{1/3}$	${{3\left(1-x_{i}\right)[-ln(1-x}_{i})]}^{2/3}$
Avarami-Erofe’ve (A4)	$[{{-ln(1-x}_{i})]}^{1/4}$	${{4\left(1-x_{i}\right)[-ln(1-x}_{i})]}^{3/4}$

**Table 2:** Estimated average kinetic and thermodynamic data using FWO and KAS model-free methods.

Sample	Model	R_2_	E_A_ [kJ·mol^−1^]	A [s^−1^]	ΔS [kJ·mol^−1^·K^−1^]	ΔH [kJ·mol^−1^]	ΔG [kJ·mol^−1^]
PFR	FWO	0.995	86.54	5.89 × 10^6^	−0.15	81.87	163.67
	KAS	0.995	81.92	1.79 × 10^6^	−0.16	77.25	166.14
50%PFR	FWO	0.975	97.84	2.92 × 10^7^	−0.15	86.32	182.24
	KAS	0.969	93.76	1.05 × 10^7^	−0.15	88.66	180.68
25%PFR	FWO	0.979	143.41	2.94 × 10^8^	−0.10	137.70	205.74
	KAS	0.976	139.79	1.30 × 10^8^	−0.11	134.08	207.04
PP	FWO	0.992	294.14	5.93 × 10^21^	0.12	288.35	203.22
	KAS	0.989	299.34	1.44× 10^22^	0.14	293.55	195.15
Overall average	FWO	0.985	155.48	1.48 × 10^21^	−0.07	148.56	188.72
	KAS	0.982	153.70	3.59 × 10^21^	−0.07	148.38	187.25

**Table 3:** CDNN model computed data.

$b_{1}=-$1.6955	$w_{1}=$ 2.1294	$w_{2}=$ −4.1525	$w_{3}=-$2.9871	$w_{4}=$ 4.3913
$b_{2}=$ 3.7023	$w_{5}=$ 0.3963	$w_{6}=$ −6.2410	$w_{7}=$ 1.7867	$w_{8}=$ 5.5891
$b_{3}=$ 2.1231	$w_{9}=$ 0.8962	$w_{10\;}=$ −4.2957	$w_{11}=$ 1.0282	$w_{12}=$ 1.9280
$b_{4}=$ 4.8091	$w_{13}=$ 0.4929	$w_{14}=$ −13.5771	$w_{15}=$ −0.8830	$w_{16}=$ −0.5733
$b_{5}=$ −4.8869	$w_{17}=$ 6.1435	$w_{18}=$ 3.1068	$w_{19}=$ 0.6498	$w_{20}=$ 17.5586
$b_{6}=$ 1.7543	$w_{21}=$ 1.6658	$w_{22}=$ 1.6902	$w_{23}=$ 8.5216	$w_{24}=-$2.7912
$b_{7\;}=$ −0.7149	$w_{25}=$ 0.6952	$w_{26}=$ 9.3220	$w_{27}=$ −12.2106	$w_{28}=$ 3.2844
$b_{8}=$ −8.8796	$w_{29}=$ 8.2967	$w_{30}=$ −1.0805	$w_{31}=$ 8.4068	
$L_{R}=$ 5 → 2	C= 0.1857	$\varepsilon_{t}=$ 0.4456	t= 13.3 h	

## Data Availability

Data are provided within the manuscript. For more information, please contact AJ Otaru (email: aotaru@kfu.edu.sa).
